# Antenatal and delivery care in rural western Kenya: the effect of training health care workers to provide "focused antenatal care"

**DOI:** 10.1186/1742-4755-7-1

**Published:** 2010-04-29

**Authors:** Peter O Ouma, Anna M van Eijk, Mary J Hamel, Evallyne S Sikuku, Frank O Odhiambo, Kaendi M Munguti, John G Ayisi, Sara B Crawford, Piet A Kager, Laurence Slutsker

**Affiliations:** 1 Kenya Medical Research Institute, Centre for Global Health Research, Kisumu, Kenya; 2 Department of Infectious Diseases, Tropical Medicine & AIDS, Academic Medical Centre, University of Amsterdam, the Netherlands; 3 Division of Parasitic Diseases, National Centre for Infectious Diseases, Centers for Disease Control and Prevention, Atlanta GA, USA; 4 University of, Nairobi, Kenya

## Abstract

**Background:**

Maternal mortality remains high in developing countries and data to monitor indicators of progress in maternal care is needed. We examined the status of maternal care before and after health care worker (HCW) training in WHO recommended Focused Antenatal Care.

**Methods:**

An initial cross-sectional survey was conducted in 2002 in Asembo and Gem in western Kenya among a representative sample of women with a recent birth. HCW training was performed in 2003 in Asembo, and a repeat survey was conducted in 2005 in both areas.

**Results:**

Antenatal clinic (ANC) attendance was similar in both areas (86%) in 2005 and not significantly different from 2002 (90%). There was no difference in place of delivery between the areas or over time. However, in 2005, more women in Asembo were delivered by a skilled assistant compared to Gem (30% vs.23%, *P *= 0.04), and this proportion increased compared to 2002 (17.6% and 16.1%, respectively). Provision of iron (82.4%), folic acid (72.0%), sulfadoxine-pyrimethamine (61.7%), and anthelminths (12.7%) had increased in Asembo compared to 2002 (2002: 53.3%, 52.8%, 20.3%, and 4.6%, respectively), and was significantly higher than in Gem in 2005 (Gem 2005: 69.7%, 47.8%, 19.8%, and 4.1%, respectively) (P < 0.05 for all). Offering of tests for sexually transmitted diseases and providing information related to maternal health was overall low (<20%) and did not differ by area. In 2005, more women rated the quality of the antenatal service in Asembo as very satisfactory compared to Gem (17% vs. 6.5%, P < 0.05).

**Conclusions:**

We observed improvements in some ANC services in the area where HCWs were trained. However, since our evaluation was carried out 2 years after three-day training, we consider any significant, sustained improvement to be remarkable.

## Background

Maternal mortality, the death of a woman while pregnant or within 42 days of termination of pregnancy, remains disturbingly high in sub-Saharan Africa. It is estimated that 270 000 maternal deaths occurred in the region in 2005 [1]. The UN millennium Development goal (MDG) on maternal health aims to reduce the number of women who die in pregnancy and childbirth by three-quarters between 1990 and 2015 [2]. To achieve this goal, it is estimated that an annual decline in maternal mortality of 5.5% is needed; however between 1990 and 2005 the annual decline was only 0.5% in the sub-Saharan region, compared to 4.2% for the middle income countries of Asia [1,3].

Maternal mortality occurs from risks attributable to pregnancy and child birth as well as from poor availability and quality of health services [4]. The most common causes of maternal mortality in sub-Saharan Africa include haemorrhage (34%), sepsis/infections (10%), hypertensive disorders (9%), HIV/AIDS (6%), and other direct causes (5%); other indirect causes contributed approximately 17% [5].

Experiences from different countries have shown that reducing maternal mortality may depend in part on the availability and use of a professional attendant at labour and delivery and a referral mechanism for obstetric care for managing complications, or the use of basic essential obstetric care facilities for all deliveries [6]. In many developing countries however, the majority of births occur at home, frequently without the help of a skilled assistant (midwife, nurse trained as midwife or a doctor) [7].

The effect of antenatal care on maternal mortality is unclear [8-10]. However, there is broad agreement that antenatal care interventions can lead to improved maternal and newborn health, which can also impact on the survival and health of the infant [11]. Additionally, the ANC visit, which many women in sub-Saharan Africa attend, is an opportunity to reach pregnant women with messages and interventions. A global evaluation of antenatal care has resulted in the recommendation to deliver antenatal services in 4 focused visits (Focussed antenatal care; FANC), one within the first trimester and 3 after quickening, and this schedule is now endorsed by WHO [12,13]. Proven effective antenatal interventions include serologic screening for syphilis, provision of malaria prevention, anti-tetanus immunization, and prevention of mother-to-child transmission of HIV [14,15]. To fully benefit from these interventions, it is important that women start visiting the antenatal clinic (ANC) early in pregnancy.

We evaluated maternal care in western Kenya in 2002 and showed that preventive interventions received at the ANC were inadequate in spite of high (90%) ANC attendance [16]. After this evaluation, the Kenyan Ministry of Health in conjunction with the Johns Hopkins Organization for International Education in Training and Reproductive Health (JHPIEGO) trained healthcare workers in FANC and malaria in pregnancy in part of the study area (Asembo). FANC emphasizes goal-oriented and women-centred care by skilled providers, whereby the quality instead of the quantity of visits is important [17]. The FANC training in 2003 emphasized identification of pre-existing health problems, early detection of danger signs arising from pregnancy, health promotion, provision of intermittent preventive treatment for malaria in pregnancy (IPTp) with sulfadoxine-pyrimethamine (SP), provision of iron and folate, birth preparedness, blood pressure measurement, growth monitoring, urine albuminuria and preparation for post-partum family planning. The training was short (3 days) and focused on need-to-know information. An interactive training approach with user-friendly materials was used. These materials enabled the providers to cascade the training to their colleagues in the place of work. Supportive supervision to reinforce skills was undertaken following the training in May-June 2003 in a random sample comprising of 25% of the health facilities in which health care workers had been trained (because of resource constraints not all health facilities received supportive supervision). The focus of the supportive supervision was to identify any gaps and to reinforce knowledge on focused antenatal care and malaria in pregnancy.

In April 2005, we conducted a repeat cross-sectional survey among a random sample of women with a recent birth living in the same areas as the previous survey to assess whether there were improvements in antenatal and delivery care and if there were differences between the area where service providers were trained in FANC and the area where training did not occur.

## Methods

The Centers for Disease Control and Prevention and the Kenya Medical Research Institute (CDC/KEMRI) conduct a demographic surveillance system (DSS) in western Kenya, since 2002. The DSS area is located in Asembo (Rarieda Division, Bondo district) and Gem (Yala and Wagai Divisions, Siaya District), of Nyanza province in western Kenya, and covers 217 villages (75 in Asembo and 142 in Gem) spread over approximately 500 km^2 ^along the shores of Lake Victoria. The vast majority of the population are members of the Luo tribe who earn their living through subsistence farming and fishing [18]. Residents of the DSS are visited in their homes every 4 months to record births, deaths, pregnancies, pregnancy outcomes, immigration and out-migration [19]. Health indicators are poor in the area when compared to national figures, with infant mortality rate estimated at 125 per 1000 live births compared to the national figure of 77 per 1000 live births, under-five mortality rate of 227 per 1000 live births compared to 115 nationally, and overall life expectancy at birth at 38 years (36 for men and 39 for women) compared to 48 nationally [19]. The maternal mortality ratio was estimated at 753 per 100,000 live births in 2003 compared to 414 per 100,000 live births nationally [20]. This area traditionally experienced intense perennial malaria transmission with an estimated entomological inoculation rate of ≈ 60-300 infectious bites per person per year [21]. However, the widespread provision of insecticide-treated nets (ITNs) during a bed net efficacy trial reduced transmission in the study area by about 90% and continuous provision of ITNs has maintained malaria transmission at a low level [22,23]. The prevalence of malaria parasitemia and anaemia was 36% and 53% respectively among pregnant women in a community survey in 2003 [24]. In the 2003 Demographic and Health Survey, the seroprevalence of HIV/AIDS in Nyanza Province (15%) was about twice as high as the national average of 7% [25]. The age-adjusted prevalence rates of HIV in men and women 13-34 years old in the DSS area were 11% and 21%, respectively (P. Amornkul, personal communication). A survey among 13 antenatal clinics in Asembo in 2005 revealed that 7 ANCs did not charge for ANC visits, and 9 provided treatments such as iron and folic acid without charge (P. Ouma, personal communication). We do not have this information for ANCs in Gem.

The sample size estimate for this study was based on a comparison of IPTp use in Asembo and Gem, and aimed to detect at least 50 percentage point difference in IPTp use in Asembo compared to Gem after FANC training, with 80% power and 95% confidence interval. Allowing for 15% failure to recruit, a random sample of 830 women was selected using a list of women who had delivered between 30^th ^of September 2004 and 30^th ^of March 2005 in the DSS [26]. Interviews were conducted by experienced interviewers in the local language using a standardized questionnaire. Participants were asked questions on ANC clinic visits, services received at the clinic, where their last delivery occurred, who assisted with the delivery and satisfaction with antenatal and delivery services. Interviewers were instructed not to probe with options. Questions were similar to the 2002 survey, except for the quality assessment of the maternal services, which had not been included in the 2002 survey.

### Data management and statistical methods

We first compared the two areas in the survey in 2005, and then compared the results of the survey in 2005 to the survey in 2002. We examined the use of antenatal and delivery care, and the type of ANC services received, and the satisfaction with the services (2005 only).

Differences in proportions were compared using the Chi-square or Fisher's exact tests as appropriate. For the comparisons of medians, we used the Wilcoxon two sample test (non-parametric). Education level was dichotomized as < 8 years or ≥ 8 years, the minimum number of years required to complete primary education in Kenya. We used Principal Components Analysis (PCA) method to generate weights for the following broad household characteristics: occupation of participant and spouse, source and quality of water, source of fuel for cooking, livestock and asset ownership, and dwelling/housing structure. The scores were used to rank the study participants in socio-economic status (SES) quintiles [27]. A medium/low SES was defined as a rank in the bottom three quintiles of the wealth index. The statistical program SAS was used for all analyses (SAS for windows version 8; SAS Institute, Cary, North Carolina, USA).

Ethical approval for this study was obtained from the institutional review boards of the Kenya Medical Research Institute (Nairobi, Kenya) and the Centers for Disease Control and Prevention (Atlanta, Georgia, USA).

## Results

### Characteristics of study participants

**Figure 1:**
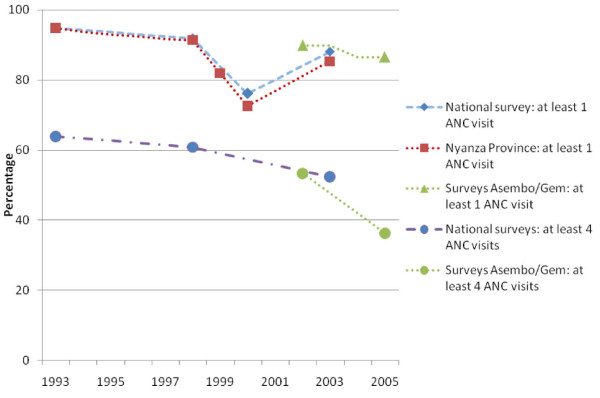
**Antenatal attendance in Kenya from 1993-2005**.

*Survey 2005*: Of the 830 women selected for the survey, 106 (12.8%) did not participate for various reasons (figure 1). A higher proportion of non-participants were younger than 20 years compared to participants (25.3% versus 14.8%, respectively, *P *= 0.01). Of 724 women who were interviewed, 702 (97.0%) were of Luo ethnicity. Median age of the women was 26 years (range 12-48 years). Compared to Gem, women from Asembo were significantly more likely to have <8 years of education and to walk shorter distances to ANC; otherwise characteristics were similar (Table 1). The median time between delivery and interview was 129 days (range 15-201), and was slightly longer for participants from Gem (135 days, range 34-201) compared to participants from Asembo (123 days, range 15-201, *P *= 0.02).

**Table 1:** Services offered in the ANC among attending women Asembo/Gem 2005 and 2002.

	Asembo 2005	Gem 2005	Asembo 2002	Gem 2002
Services	n (%)	n (%)	n (%)	n (%)
Palpation of abdomen	238/241 (98.8)	381/384 (99.2)	290/292 (99.3)	272/278 (97.8)
Listen to foetal heart rate	233/241 (96.7)	377/384 (98.2)	NA	NA
Tetanus vaccination	232/242 (95.9)	361/382 (94.5)	282/292 (96.6)	273/278 (98.2)
Weight measurement	191/241 (79.3)*	377/384 (98.2)	241/293 (82.3)*	277/278 (99.6)
Iron supplementation	197/239 (82.4)*	260/373 (69.7)	154/289 (53.3)	149/277 (53.8)
Folic acid supplementation	167/232 (72.0)*	170/356 (47.8)	153/290 (52.8)*	100/269 (37.2)
SP ≥ 1 dose	148/240 (61.7)*	74/373 (19.8)	58/286 (20.3)	63/277 (22.7)
SP ≥ 2 doses	67/237 (28.3)*	14/370 (3.8)	22/237 (9.3)	20/357 (5.6)
Blood pressure measurement	152/240 (63.3)*	274/384 (71.4)	198/292 (67.8)	187/276 (67.8)
Test haemoglobin level	72/237 (30.8)	144/377 (38.2)	148/286 (51.8)	138/274 (48.5)
Urine analysis done	73/240 (30.4)*	88/383 (23.0)	59/293 (20.1)*	30/278 (10.8)
Stool test	58/240 (24.2)*	45/384 (11.7)	50/293 (17.1)*	22/278 (7.9)
Antihelminth treatment	30/237 (12.7)*	15/365 (4.1)	13/283 (4.6)*	2/268 (0.8)
Offered test for STD (e.g. syphilis)	20/232 (8.6)	45/377 (11.9)	62/289 (21.5)	49/277 (17.7)
Offered test for HIV	26/232 (11.2)*	139/379 (36.7)	NA	NA
Health talk provided	42/240 (17.5)*	115/382 (30.1)	45/289 (15.6)	35/278 (12.6)
Information provided on:				
Danger signs in pregnancy	25/241 (10.4)	44/376 (11.7)	49/291 (16.8)	33/276 (12.0)
Malaria in pregnancy	58/241 (24.0)	83/381 (21.8)	NA	NA
Birth plan discussed	33/241 (13.7)*	80/380 (21.1)	NA	NA
Danger signs during delivery	17/241 (7.1)	37/380 (9.7)	NA	NA
Danger signs after delivery	11/240 (4.6)	33/381 (8.7)	NA	NA
Breastfeeding	36/240 (15.0)*	85/383 (22.2)	NA	NA
Family planning	18/240 (7.5)*	97/383 (25.3)	NA	NA

NA: Not Available SP: sulfadoxine-pyrimethamine

* p < 0.05 comparing areas in the same survey

*Survey 2005 vs. 2002*: In 2002, participants in Asembo were less likely to have <8 years of education than in 2005, and had a higher SES compared to participants from Gem. Other characteristics were similar to 2005. Overall in both areas, more women reported they used a bus or bicycle to visit the nearest antenatal clinic in 2005 than in 2002 (14% vs. 7%, *P *< 0.01).

### Use of maternal care in Asembo and Gem

*Survey 2005*: In 2005, ANC attendance was similar between the two areas (overall 86%, Table 2), with a median number of 3 ANC visits among attending women. More women in Asembo started attending in the second trimester compared to Gem (64% vs. 53%, respectively, *P *< 0.01). More women in Asembo than in Gem delivered in a health facility (20% vs. 14%, *P *= 0.05), and delivered with the assistance of a skilled attendant (30% vs. 23%, *P *= 0.04). Fewer women in Asembo compared to Gem delivered unattended (12% vs. 18%, *P *= 0.03). Traditional birth attendants (TBAs) were more frequently used in Asembo (36.8%) compared to Gem (26.8%, *P *= 0.006).

*Survey 2005 vs. 2002*: Overall, ANC attendance was slightly lower in 2005 compared to 2002 (86% vs. 90%, *P *= 0.06). Additionally, the total number of visits had decreased (≥ 4 visits: 36% in 2005 vs. 53% in 2002, *P *< 0.01), and women started attending later (36% of women started in the third trimester in 2005 vs. 23% in 2002, *P *< 0.01). Compared to 2002, there was no difference in place of delivery in Asembo; however, more women delivered with the assistance of a skilled attendant in Asembo in 2005 compared to Asembo or Gem in 2002 (both *P *< 0.01) (Table 2). Overall, there was no statistical difference in number of women who delivered unattended between the surveys (17.8% in 2002 and 16.0% in 2005, both areas combined).

### Services offered at the ANC

*Survey 2005*: Palpation of the abdomen, listening to the foetal heart, tetanus vaccination, weight measurement and iron supplementation comprised the bulk of services offered in 2005 (Table 3). Weight and blood pressure measurements, health talks and offering of HIV tests were done less often in Asembo compared to Gem, whereas the provision of iron, folic acid, SP, stool test and urine analysis were done more often in Asembo. Provision of health education was low in both areas (range 5-25%); the discussion of a birth plan, and information on breastfeeding and family planning were less frequently mentioned in Asembo compared to Gem.

*Survey 2005 vs. 2002*: Iron and folic acid supplementation increased in both areas significantly in 2005 compared to 2002, but relatively more so in Asembo (Table 3). Provision of SP increased only in Asembo in 2005 compared to 2002. The provision of haemoglobin tests significantly decreased in both areas in 2005 compared to 2002, whereas the provision of urine analysis tests significantly increased. Antihelminthic treatment, although modest overall, increased significantly in both areas but relatively more so in Asembo. Offering of tests for sexually transmitted diseases other than HIV decreased significantly in both areas (overall 11% in 2005 vs. 20% in 2002, *P *< 0.01, with no difference by area). The provision of health talks increased significantly among ANC attendees only in Gem in 2005 compared to 2002 (*P *< 0.01).

### Satisfaction with antenatal and delivery care in 2005

We explored differences in the level of satisfaction of women who attended the ANC or delivered in a health facility in the two areas in 2005 as measured by the indicators in Table 4. The majority of women waited more than an hour in both areas (Asembo 52.4% vs. Gem 59.9%, *P *= 0.08). The median waiting time at delivery was 10 minutes (range 0-240 minutes), with no difference between Asembo and Gem (median of 5 minutes, range 0-65 minutes, and median of 10 minutes, range 0-240 minutes, respectively, *P *= 0.07). In the ANC, significantly more attendees from Asembo thought service providers explained procedures and encouraged questions compared to attendees from Gem. Overall, more women in Asembo rated the antenatal service as very satisfactory (17.0%) compared to Gem (6.5%, P < 0.001) The rating of the delivery services in a health facility among the 119 women who made use of it was low, with only one woman reporting it as very satisfactory and the majority of women rating it as average (68.9%).

## Discussion

Training in focussed antenatal care did not seem to change the use of antenatal care but may have led to improvement of some specific services (provision of preventive treatment such as iron, folic acid, and SP), a higher rating of the quality of care offered in the ANC and an increase in the number of deliveries with a skilled attendant. However, there was no improvement in other important services such as the provision of information.

Recall bias due to the time passed between last antenatal contact and time of interview is a major limitation to this survey. We did not have information on the date of the last antenatal visit in the 2^nd ^survey, but the median time between delivery and the interview was 129 days (range 15-201 days). We were able to validate some verbal responses with information from ANC cards; there was over 90% agreement with regards to questions on number of antenatal visits, tetanus injections, and iron and folate supplementation. The agreement with regards to questions on malaria treatment and prevention was around 80%.

Another major limitation was the time passed between the training of antenatal staff and the conduct of the survey. We did not visit the antenatal clinics during the period of the second survey to confirm if the staff trained in 2003 had remained in the clinic. Health facility staff changes are common in Kenya, and there is a chronic lack of human resources, resulting in the overburdening of the staff who are present [28]. In this respect, it may be encouraging that we did find any difference at all, given the short duration of the training (3 days) and the time passed between training and second survey. Additionally, not all differences detected may have been due to the training; e.g. differences in ANC costs and availability of drugs and tests by study area may be an alternative factor affecting our findings. Unfortunately, we do not have information on this available for the whole study area.

Although the proportion of women who visited the ANC at least once was not different between the study areas and only decreased slightly between the different surveys, the number of visits per woman had decreased over time in both areas. Significantly fewer women had visited 4 or more times, the recommended number of ANC visits, during the last survey. The reason for this is not clear; level of education, distance to antenatal clinic and parity are known to affect ANC attendance [29,30], but there was no difference in these characteristics between the surveys to explain the decrease in visits. In the Kenya 2003 Demographic and Health Survey, 88.1% of the women visited ANC at least once and the proportion of women who visited at least 4 times was 52.3%, a figure comparable to our 2002 proportion of 53.4% (Figure 1) [25]. There are no recent national survey data available to assess if our finding of a decline in the proportion of women visiting at least 4 times in 2005 indicates a national or a local trend. Past national surveys have shown a tendency to decreased attendance, as presented in figure 1.

No difference was seen in place of delivery between the surveys and areas; in both areas the majority of deliveries occurred at home or, less frequently, in the home of a TBA. However, an increase in deliveries with a skilled attendant was seen in both areas in 2005, but relatively more in Asembo. A skilled attendant at delivery is one of the MDG indicators, and is considered one of the primary means to reduce maternal mortality [7]. Therefore, this increase is encouraging. Nonetheless, 7-8 women out of 10 delivered in the absence of professional help. TBAs were more frequently used in Asembo compared to Gem, but only assisted in 27% to 37% of deliveries in these areas. There has been debate on the impact of TBAs on maternal mortality [8,31]; systematic reviews showed that TBA training appeared to increase antenatal care attendance, but no improvements in maternal mortality could be detected [32,33]. Their role for delivery care in our study area seems to be limited to about one third of the deliveries; however, this is still more than the number of women assisted by a skilled attendant or in a health facility in the study area. TBAs could still be important for community activities such as ensuring that women deliver with skilled assistants, accompanying women to delivery units, and reminding women of ANC visits.

Women rated their satisfaction with ANC services modestly higher in Asembo compared to Gem. However, the overall satisfaction rating for health services provided during a health facility delivery was low. The percentage of women delivering in a health facility was 17%, which was lower than the provincial estimate of 36.2% in 2003, lower than the estimates in other rural Provinces of Kenya (range 28.4%-66.9%, except for the North Eastern province where it was 7.7%), the Kenya national average (40.1%) [25], or rural areas in other countries in the region, such as Tanzania (38.8.%) or Uganda (36.3%) [34,35]. Client satisfaction is known to affect health seeking behaviour; a recent Tanzanian study showed that women preferred to pay for deliveries in private health facilities with perceived better quality services instead of government health facilities with free delivery services that were closer to their homes [36]. If more women are to deliver in health units, more needs to be done to improve the expense and service.

Of all the differences noted in antenatal services which could be attributed to training, the increases in IPT doses and iron and folate supplementation were the highest [26]. However, effect of training on other services was disappointing, particularly with regards to the provision of information. Despite training, HCWs in ANCs in Asembo did not perform better than Gem, and in some areas (e.g. discussing a birth plan, information on breastfeeding and family planning), HCWs in Asembo performed worse. This indicates that only some parts of the training were incorporated in the daily routine, but that others may have been ignored. Alternatively, HCWs in Gem may have received training that we were not aware of, or some of the persons trained in Asembo may not have used or cascaded their new skills for various reasons, *e.g. *the high work load in the ANC due to understaffing or the person who has been trained may not be working in the ANC (J. Ayisi, personal communication). Further exploration is needed to learn how to improve uptake of all key components of the training. The package of focused antenatal care is condensed [17,37], and given all the information which needs to be provided in each visit, particularly the first visit which includes HIV-testing and counselling, not all of the information given may be adequately heard and understood [38]. In addition, lectures by ANC staff may not the best means of health education [39]. Alternative strategies for some aspects of health education might improve delivery and retention of key messages; options to be explored include videos while waiting for the ANC visit, or fliers for family discussion. Moreover, TBAs and other community resource persons could assist in making pregnant women and their families aware of important issues such as danger signs, formulating a birth plan, malaria prevention, and breastfeeding, during home visits or group meetings to further emphasize key messages provided during ANC visits. In Nepal, community mobilisation using women's group meetings with a facilitator from the community was associated with a decrease in maternal mortality [40]. A trial in Burkina Faso using female community leaders to promote health messages related to reproductive health resulted in an increase in antenatal visits and an earlier start of visits [41]. Thus, training of health care workers and mobilization of communities may lead to better health outcomes.

The number of women who reported being tested for syphilis decreased in both areas over time, and this is a worrying trend. Detecting and treating syphilis in pregnancy is a proven effective important prevention strategy for decreasing birth outcomes such as stillbirths and low birth weight [42]. It is not clear if this decline was due to lack of test kits in Kenya during the time of the second survey in 2005. However, an evaluation in Kenya of focused antenatal care from the perspective of the healthcare provider in a neighbouring area concluded that many clinics were not properly prepared to offer the complete package, including a lack of tests for syphilis, haemoglobin, urine and HIV and the lack of essential equipment [37] (J. Ayisi, personal communication). HIV testing was not evaluated in our survey in 2002. However, during 2005, HIV programmes were being implemented in the antenatal clinics in this area. Subsequent surveys will be able to assess progress.

A recent autopsy study from Mozambique showed that the majority of maternal deaths were from infectious causes, and approximately 40% from obstetric causes [43]. The relative larger contribution of infectious causes in Africa was also reflected in the review by Khan et al [5]. Given this information, it may be worth reconsidering if a different focus or strategy of antenatal (and obstetric) care may assist in the prevention of these deaths. Currently the bulk of work in the antenatal clinic is weight measurements, palpation of the abdomen, listening of the foetal heart and tetanus vaccination. Some of these activities have not been shown to improve maternal health (e.g. weight measurement), whereas other preventive strategies which have been proven effective are used infrequently, (e.g. early identification of HIV and syphilis infection) [10]. In addition, there are other preventive strategies such as micronutrient supplementation, provision of calcium tablets, and helminth and antibiotic treatment in 2^nd ^or 3^rd ^trimester which may be particularly useful in Africa [44]. Evaluations of components of antenatal care are often conducted in the industrialized countries where antenatal needs, possibilities, opportunities and social context are different [45]. A more rigorous evaluation of antenatal components with regards to the local circumstances in sub-Saharan Africa may lead to antenatal procedures which are more effective, practical, and feasible for the improvement of maternal health, and possibly the reduction of maternal mortality.

## Conclusions

The training of antenatal staff in Asembo was associated with improvements in performance but the difference with the untrained clinics of Gem was modest. Overall, services delivered remained sub-optimal, especially with regards to providing key information to pregnant mothers and ordering essential tests. However, the improvement detected is encouraging given the short duration of the training and the time lapse between training and evaluation. An antenatal clinic training program may need to include an evaluation among the attendees over longer periods of time, to assess which components of the training may need further attention. To meet international goals to reduce maternal mortality in developing countries, alternative methods to improve antenatal and delivery care should be explored.

## Competing interests

The authors declare that they have no competing interests.

## Authors' contributions

PO, AMvE, MH and KM conceived of the study, and participated in its coordination. All authors participated in the design. PO, ES, FO, and JA carried out the study. SC assisted in the analysis and data interpretation. PO and AMvE drafted the manuscript. All authors read and approved the final manuscript.
